# Do Alterations in Mitochondrial DNA Play a Role in Breast Carcinogenesis?

**DOI:** 10.1155/2010/604304

**Published:** 2010-06-06

**Authors:** Thomas E. Rohan, Lee-Jun Wong, Tao Wang, Jonathan Haines, Geoffrey C. Kabat

**Affiliations:** ^1^Department of Epidemiology and Population Health, Albert Einstein College of Medicine, 1300 Morris Park Avenue, Bronx,NY 10461, USA; ^2^Department of Molecular and Human Genetics, Baylor College of Medicine, One Baylor Plaza, Houston, TX 77030, USA; ^3^Department of Molecular Physiology & Biophysics, Vanderbilt University Medical Center, 519 Light Hall, Nashville, TN 37232-0700, USA

## Abstract

A considerable body of evidence supports a role for oxidative stress in breast carcinogenesis. Due to their role in producing energy via oxidative phosphorylation, the mitochondria are a major source of production of reactive oxygen species, which may damage DNA. The mitochondrial genome may be particularly susceptible to oxidative damage leading to mitochondrial dysfunction. Genetic variants in mtDNA and nuclear DNA may also contribute to mitochondrial dysfunction. In this review, we address the role of alterations in mtDNA in the etiology of breast cancer. Several studies have shown a relatively high frequency of mtDNA mutations in breast tumor tissue in comparison with mutations in normal breast tissue. To date, several studies have examined the association of genetic variants in mtDNA and breast cancer risk. The G10398A mtDNA polymorphism has received the most attention and has been shown to be associated with increased risk in some studies. Other variants have generally been examined in only one or two studies. Genome-wide association studies may help identify new mtDNA variants which modify breast cancer risk. In addition to assessing the main effects of specific variants, gene-gene and gene-environment interactions are likely to explain a greater proportion of the variability in breast cancer risk.

## 1. Introduction

Breast cancer has a complex, multifactorial etiology, with contributions from both genetic and environmental factors. Although its etiology is incompletely understood, it has been estimated from studies of twins that hereditary factors explain about 27% of the variation in breast cancer risk, with the remainder being due to nonshared environmental and lifestyle factors [1]. Factors that have been associated with increased risk include increasing age, a history of breast cancer in a first-degree relative [2, 3], a history of benign breast disease [4, 5], menstrual and reproductive factors [6–8], use of hormone therapy [9, 10], a relatively high body mass index (BMI) (in postmenopausal women) [11], alcohol consumption [12], and possibly cigarette consumption [13], while physical activity has been associated with reduced breast cancer risk [14]. Dietary factors (e.g., a relatively high fat intake and relatively low fruit and vegetable intake) have also been postulated to play a role in the etiology of breast cancer [15–17], although the epidemiologic evidence for this is not consistent. Collectively, the generally accepted risk factors for breast cancer explain perhaps 40% of the variation in breast cancer incidence [18]. 

There is now a considerable body of evidence to support a role for oxidative stress in carcinogenesis [19]. Oxidative stress is a disturbance in the balance between the production of reactive species (RS) (including reactive oxygen species (ROS)) and antioxidant defenses, resulting in a relative excess of RS [19–21]. RS are unstable and can react with and damage nuclear and mitochondrial DNA [22]. Additionally, they may alter the expression of genes that stimulate cell proliferation and differentiation [22, 23], and cause lipid peroxidation, protein modification, and membrane disruption [18]. 

Due to their role in producing energy via oxidative phosphorylation, the mitochondria are a major source of production of reactive oxygen species (ROS) [24, 25]. Furthermore, mitochondrial DNA (mtDNA) may be particularly vulnerable to oxidative damage because they lack protective histones and the efficient DNA repair mechanisms present in the nucleus [24, 26, 27]. Indeed, the mutation rate of mtDNA has been reported to be 10–200 times that of nuclear DNA [28–30]. Damage to mtDNA due to ROS may provide (at least in part) a mechanistic explanation for the association with breast cancer of many of the risk factors described above [18]. For example, risk of breast cancer related to reproductive and hormonal factors could be due to the metabolism of estradiol to reactive hydroxy radicals through redox cycling of the catechol estrogens [18, 31], while alcohol metabolism might also result in the generation of ROS [32]. 

A possible role of the mitochondria in cancer was first postulated by Warburg 70 years ago [33], and most research has focused on somatic mutations in mtDNA [34, 35]. Recently, however, a number of studies have addressed the possibility that mitochondrial DNA variants may also play a role in the etiology of specific cancers [24, 35, 36]. In this review, we summarize what is known about the role of oxidative stress in relation to cancer generally and to breast cancer in particular, how exogenous and endogenous exposures might contribute to oxidative stress, the function of the mitochondrion and the mitochondrial genome, and the possible role of mtDNA mutations and polymorphisms in breast carcinogenesis. Finally, we discuss specific topics for future research.

## 2. The Mitochondrion

Mitochondria are the energy-transducing organelles of eukaryotic cells in which fuels that drive cellular metabolism (e.g., carbohydrates and fats) are converted into adenosine triphosphate (ATP) through the electron transport chain and the oxidative phosphorylation system (the “respiratory chain”) [37, 38]. They are also involved in calcium buffering and the regulation of apoptosis [39]. They arose as intracellular symbionts in the evolutionary past, and can be traced to the prokaryote *α*-proteobacterium [40]. There are hundreds to thousands of mitochondria per cell [37]. 

**Figure 1:**
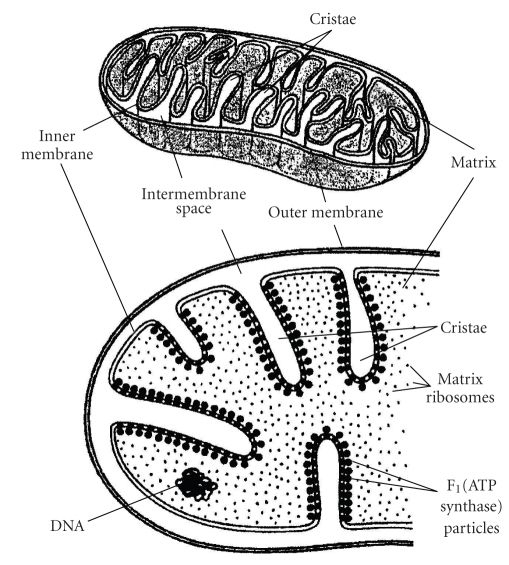
Mitochondrial structure adapted by Freitas [41] (Reproduced with permission from Freitas Nanomedicine, Volume I: Basic Capabilities. Austin: Landes Bioscience, 1999:264), Vander et al. (Reproduced with permission of The McGraw-Hill Companies) [42], and Becker, Deamer (Used by permission of Pearson Education, Inc.) [43].

Structurally, mitochondria have four compartments: the outer membrane, the inner membrane, the intermembrane space, and the matrix (the region inside the inner membrane) (Figure 1) [37, 41]. The respiratory chain is located in the inner mitochondrial membrane. It consists of five multimeric protein complexes: reduced nicotinamide adenine dinucleotide (NADH) dehydrogenase-ubiquinone oxidoreductase (complex I), succinate dehydrogenase-ubiquinone oxidoreductase (complex II), ubiquinone-cytochrome c oxido-reductase (complex III), cytochrome c oxidase (complex IV), and ATP synthase (complex V). In addition, the respiratory chain requires 2 small electron carriers, ubiquinone and cytochrome c.

Energy generation via ATP synthesis involves two coordinated processes [37]: electrons (hydrogen ions derived from NADH and reduced flavin adenine dinucleotide) are transported along the complexes to molecular oxygen, resulting in the production of water; simultaneously, protons are pumped across the mitochondrial inner membrane (from the matrix to the intermembrane space) by complexes I, III, and IV. ATP is generated by the influx of these protons back into the mitochondrial matrix through complex V (ATP synthase).

## 3. Oxidative Stress

Oxidative stress arises when there is an imbalance between the production of reactive species (RS) and antioxidant defenses in favor of the former, resulting in an increase in cellular levels of RS [19, 22]. RS are molecular entities that include reactive oxygen species (ROS), such as superoxide anion, hydrogen peroxide, and the hydroxyl radical, reactive nitrogen species (RNS), including the radicals nitric oxide and nitrogen oxide, as well as reactive halogen and sulfur species. They possess one or more unpaired electrons, thereby rendering them inherently unstable [19, 20, 44]. 

ROS, the most extensively studied of the reactive species, are highly reactive molecules or molecular fragments that are continually produced in all aerobic organisms, mostly as a consequence of aerobic respiration and oxidative phosphorylation [45]. The close proximity of mtDNA to the site of ROS production makes it more susceptible to oxidative damage and may explain the high frequency of mtDNA mutations seen in cancer [46]. 

ROS have physiological roles in a number of cellular processes, including effects on vascular tone and platelet adhesion, and, importantly, on intracellular and intercellular signaling [45] (e.g., H_2_O_2_ is a key intracellular messenger at subtoxic levels in signaling pathways involving epidermal growth factor and PI 3-kinases [47, 48]) and induction of apoptosis and senescence [49, 50]. As mentioned earlier, the mitochondria are a major source of ROS production [24, 25]. Specifically, during mitochondrial oxidative metabolism and ATP synthesis, the majority of the oxygen consumed is reduced to water. However, about 1%–5% of molecular oxygen is converted to ROS, primarily superoxide anion, which is formed by an initial one-electron reduction of molecular oxygen [22, 45]. Superoxide can be dismutated by superoxide dismutase to yield hydrogen peroxide. In the presence of partially reduced metal ions, hydrogen peroxide is converted through Fenton and Haber-Weiss reactions to a hydroxyl radical, which is highly reactive and can interact with nucleic acids, lipids, and proteins [44]. Other endogenous sources of ROS production include neutrophils, eosinophils, macrophages, and peroxisomes. ROS can be produced not only as a result of endogenous cellular processes, but also in response to exogenous exposures. Exogenous sources of ROS production include chlorinated compounds, radiation, metal ions, some peroxisome proliferating compounds, hormone therapy, cigarette smoke, and ethanol [22, 51].

Antioxidant defenses operate through cellular, membrane, and extracellular mechanisms [44]. Cellular mechanisms include the dismutase, peroxidase, and catalase enzymes; additionally, intracellular ROS production is decreased by the ability of mitochondrial cytochrome oxidase to function catalytically in the electron transport chain without releasing ROS [52]. Cell membrane defenses include antioxidants such as vitamin E, beta-carotene, and coenzyme Q; furthermore, membrane structure is important, in that resistance to antioxidant attack is enhanced by having appropriate proportions of cholesterol and phospholipids. Extracellular antioxidant defenses include the metal-binding proteins (e.g., transferrin), low molecular weight molecules such as bilirubin and vitamin C, and extracellular forms of glutathione peroxidases and superoxide dismutases [44].

## 4. Oxidative Stress and Cancer

There is now substantial evidence to suggest that relative increases in reactive species in the cell, either as a result of physiological modification or through exogenous exposures, contribute to carcinogenesis [19, 22, 45]. There are a number of possible mechanisms through which this might occur. As mentioned earlier, RS can directly damage DNA. For example, the hydroxyl radical may activate oncogenes or inactivate tumor suppressor genes through point mutations, activate chemical carcinogens, and prevent DNA repair [45]. RS might also stimulate the expansion of initiated cell clones through modulation of genes related to apoptosis or proliferation, with resultant stimulation of cell proliferation and suppression of apoptosis [19, 45]. In addition, the effects of RS may be influenced by polymorphisms in genes involved in carcinogen metabolism, antioxidant defenses, and DNA repair [35].

## 5. Oxidative Stress and Breast Cancer

Several lines of evidence provide support for a role of oxidative stress in the etiology of breast cancer [18]. Markers of oxidative damage, such as DNA adducts and lipid peroxidation products (e.g., DNA-malondialdehyde (MDA) adducts, 8-oxo-7,8-dihydro-2′-deoxyguanosine (8-oxodG)), can be detected in breast tissue, and several relatively small clinical studies have mostly shown that levels of such markers are higher in breast cancer tissue [53–57] and in adjacent normal tissue from breast cancer cases [55, 58, 59] than in breast tissue from those without breast cancer, although two studies have shown the reverse [60, 61]. In cross-sectional studies, higher levels of oxidative damage markers have also been observed in the serum/plasma [62–69] and urine [70] of breast cancer cases than those of controls, albeit not consistently [71, 72]. In the only cohort study to date, urinary 15-F_2t_-IsoP and 15-F_2t_-IsoPM levels (markers of oxidative stress) were associated with increased risk of postmenopausal breast cancer in women with relatively high BMIs (≥25 kg/m^2^) [73]. Also, urinary MDA excretion has been observed to be higher in women with relatively high breast density (indicative of increased breast cancer risk [74]) than in those with less dense breast tissue [75]. In contrast, in one study, MDA levels in nipple aspirate fluid were shown not to differ between breast cancer cases and controls, whereas levels of 8-iso-PGF_2*α*_, another marker of oxidative stress, were shown to be lower in cases than in controls [76].

## 6. The Mitochondrial Genome

**Figure 2:**
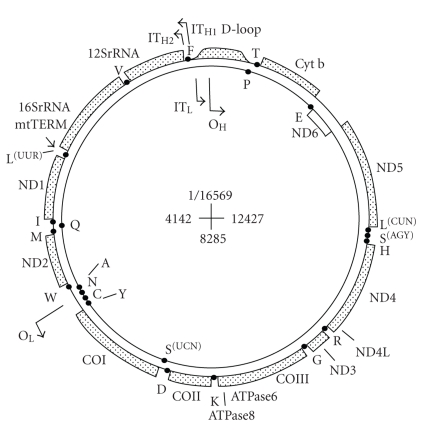
See work by Taanman in [38]. The light (L) strand encodes for eight tRNAs and a single polypeptide. The 13 protein products are subunits of the enzyme complexes of the respiratory chain/oxidative phosphorylation system (DiMauro and Schon, 2003) [37]. Mammalian mtDNA does not have introns, and has only a few intergenic sequences. The displacement loop (D-loop) region is a short, three-stranded structure in which a short nucleic acid strand, complementary to the L-strand, displaces the H-strand. The D-loop is the major control site for mtDNA expression, containing the leading-strand origin of replication and the major promoters for transcription [38].

Mitochondria contain their own genome, mitochondrial DNA (mtDNA), which is transmitted through the female germline [77]. MtDNA is located in the mitochondrial matrix and is present in multiple copies per mitochondrion [38, 77]. The human mitochondrial genome is a closed, double-stranded DNA molecule of 16,569 bp, which contains 37 genes. Most of the genes are located on the heavy (H) strand, which encodes for two ribosomal RNAs, 14 transfer RNAs (tRNAs), and 12 polypeptides (Figure 2) [38]. The light (L) strand encodes for eight tRNAs and a single polypeptide. The 13 protein products are subunits of the enzyme complexes of the respiratory chain/oxidative phosphorylation system [37]. Mammalian mtDNA does not have introns, and has only a few intergenic sequences. The displacement-loop (D-loop) region is a short, three-stranded structure in which a short nucleic acid strand, complementary to the L-strand, displaces the H-strand. The D-loop is the major control site for mtDNA expression, containing the leading-strand origin of replication and the major promoters for transcription [38]. 

## 7. The Mitochondrial Genome and Cancer

The mitochondria are not only a major source of ROS production, but, as mentioned earlier, they are also particularly susceptible to damage by ROS because the mitochondrial genome is close to the site of ROS production, lacks histones and introns, and has much less efficient DNA repair mechanisms than nuclear DNA [24, 26, 27, 46]. Given the roles of the mitochondria in energy metabolism, generation of reactive oxygen species, aging, and the initiation of apoptosis, mitochondrial damage could contribute to carcinogenesis by causing dysfunctional mitochondrial-induced apoptosis and driving cellular proliferation [78–80]. 

During cell division, mitochondria segregate randomly among daughter cells [77]. In normal tissues, all copies of mtDNA are identical (homoplasmy). When pathogenic mutations of mtDNA arise, they usually affect some but not all mtDNAs within a cell. Hence, the affected cells (and associated tissues) will contain an admixture of mutant and wild-type mtDNAs, a situation referred to as heteroplasmy. In cancer cells, however, due to clonal expansion most somatic mtDNA mutations appear to be homoplasmic [81].

## 8. Alterations in the Mitochondrial Genome and Breast Cancer

Experimentally, depletion of mtDNA-encoded oxidative phosphorylation genes has been shown to result in tumorigenic transformation of breast epithelial cells [82]. In humans, several studies have shown a relatively high frequency of mtDNA mutations in breast tumor tissue (range 20%–93%) [83–88], although the higher estimates may be due partly to sample contamination [87]. Furthermore, a recent study suggested that mtDNA D-loop Mn*l*II site mutations might be associated with increased breast cancer risk [89], and two studies have demonstrated breast cancer-specific deletions in mtDNA [88, 90].

In addition to somatic changes, mtDNA variants (polymorphisms) may have subtle effects on ROS production, and it has been postulated that if the variant reduces the efficiency of mitochondrial functioning, the accumulation of ROS may affect cancer risk [91]. Hence, several studies have examined the association between mtDNA variants and breast cancer risk [24, 36, 91–98] (Table 1), but their results do not allow clear conclusions to be drawn regarding specific associations. The G10398A mtDNA polymorphism has received the most attention, and breast cancer risk in association with the 10398A allele has been shown to be associated with increased risk in African-Americans [24], Caucasians [91], and East Indians [94] in some studies; not associated with altered risk in either African-Americans [97] or in Caucasians [95, 96] in other studies; and associated with decreased risk in one Caucasian (Polish) population (reported as risk in association with A10398G) [93]. The remaining investigations have shown no association with the mtDNA D-loop (CA)_n_ repeat polymorphism in Chinese [98] or with a range of variants in a Spanish population [95]. Bai et al. [91] examined risk in association with 69 mtDNA variants and observed a few that were associated with altered risk. Most studies to date have been relatively small [91, 93–95] and none has undertaken a genome-wide approach, although the study of Bai et al. [91] did focus on variants in the rRNA, mRNA, tRNA, and D-loop regions of mtDNA. Only one study [24] has involved a two-stage approach of first identifying a possible association and then testing it in an independent sample. 

It is conceivable that mtDNA variants might act jointly to influence breast cancer risk. Furthermore, several factors involved or potentially involved in the etiology of breast cancer—estrogens, cigarette smoking, alcohol consumption, and caloric intake—might modify mitochondrial function. Investigation of interactions of variants with each other and with environmental exposures is warranted because once patterns of association and interaction are understood, the effects of specific genes and environmental exposures on phenotype may be estimated more accurately [99]. In this regard, two studies have investigated interactions between mtDNA variants and breast cancer risk [36, 92]. In a relatively large study Canter et al. [92] reported a significant interaction between G10398A and T4216C in relation to breast cancer risk in African-American women. In a smaller study, Covarrubias et al. [36] evaluated associations between breast cancer risk and two-way interactions between 17 mtDNA variants. One interaction, between variants 12308G and 10398G, was highly statistically significant, suggesting that these variants increase a woman's risk of breast cancer by a factor of 3.

In relation to exogenous exposures, susceptibility to the effects of mitochondrial dysfunction may be particularly important in estrogen-related cancers such as breast cancer, because the normal metabolism of estradiol through redox-cycling intermediates may also generate local ROS and oxidative injury in the breast, thereby predisposing to neoplastic transformation [24]. Furthermore, mitochondrial transcription is enhanced by estrogen treatment, suggesting that estrogen-induced mitochondrial transcription is likely to contribute to breast carcinogenesis [51, 100]. Smoking-related damage to respiratory chain function in lymphocytes has been correlated with measures of oxidative damage [101, 102], and smoking-related oxidative damage has been shown to inhibit mitochondrial enzyme activity in platelets and to cause mitochondrial dysfunction in alveolar macrophages [103]. Two recent studies have shown that cigarette smoking is associated with an increase in mitochondrial DNA copy number [101, 104], which might represent a compensatory response to smoking-induced oxidative damage [104]. Shen et al. [105] found that mtDNA copy number had a significant positive association with risk of breast cancer and a significant inverse association with several important endogenous antioxidants (glutathione, CuZnSOD activity, and catalase) and the prooxidant myeloperoxidase, suggesting that mtDNA copy number may be associated with breast cancer risk, possibly through an oxidative stress mechanism. 

Alcohol consumption is associated with the generation of ROS [18], and a recent study showed that alcohol consumption was associated with an increase in breast cancer risk only in those with the G allele of the A10398G polymorphism [96]. Another recent study [106] found no difference in the frequency of mtDNA mutations by alcohol intake, dietary intake, or MnSOD genotype [106]. 

Finally, the mitochondria use oxidative phosphorylation to convert dietary calories into usable energy, and they generate ROS as a toxic by-product. Hence, it has been proposed that interaction between dietary caloric intake at modern levels (in conjunction with a sedentary lifestyle) and mtDNA polymorphisms favorable for selective adaptation to cold Northern climates during human evolution might influence disease risk [79]. In this regard, it is of interest that insulin-resistant individuals have been observed to have defects in mitochondrial content, structure, and function, with possible consequences for mitochondrial energetics [107]. 

**Figure 3:**
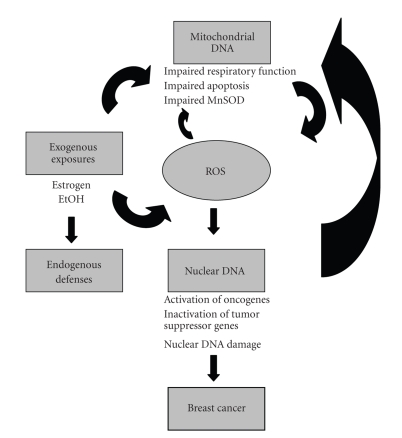
Schema showing how ROS may affect mitochondrial and nuclear DNA leading to breast carcinogenesis.

Figure 3 presents a schema for how exogenous exposures, endogenous defences, and mtDNA variants might influence ROS production, subsequent DNA damage, and breast cancer risk. 

## 9. Functional Studies of Mitochondrial Changes in Cancer Cells

The functional significance of germline variants and somatic mtDNA alterations with respect to cancer development is not well understood. However, it is clear that not all mtDNA alterations or germline variants are functionally significant [85, 108–112]. Indeed, the majority of the somatic mtDNA alterations identified so far does not have clear pathogenic roles, and may simply represent the consequences of genomic instability and oxidative DNA damage during the multistep carcinogenic process [110]. However, a small number of alterations may allow a selective growth advantage for tumorigenesis [113] or may initiate cross-talk with the nucleus, thereby altering expression of genes involved in energy metabolism and tumorigenesis [110]. The mtDNA G10398A polymorphism, which results in the substitution of threonine for alanine within the NADH dehydrogenase (ND3) subunit of Complex I and has been associated with increased risk of breast cancer in African-Americans in some studies, may lead to increased ROS production [24]. Although the effect of each individual alteration or variant may be subtle, cumulatively such changes may have functional consequences.

## 10. Recommendations for Further Research

To date, few studies have examined the association of genetic variants or somatic mutations in mitochondrial DNA with the risk of breast cancer. In addition, few studies have investigated risk in association with interactions between specific genetic variants, or with interactions between genetic variants and established breast cancer risk factors (e.g., alcohol consumption and hormone therapy). Below we describe several promising directions for exploring the role of mitochondrial DNA in the development of breast cancer.

## 11. Genome-Wide Association Studies

Disease-associated mutations in high-(*BRCA1/2*) or moderate-risk (*TP53, PTEN, LKB1, CDH1, ATM, RAD50, CHEK2*) susceptibility genes identified to date explain only 25% of the familial aggregation of breast cancers and only a smaller proportion (~5%–10%) of sporadic disease [3, 114, 115]. Thus it is clear that there must be other candidate genes that contribute modestly to risk [116]. Genome-wide association studies (GWAS) represent one approach to the identification of such genes, and their conduct has been facilitated by the development of the HapMap, a genome-wide database of patterns of human genetic variation [99, 117]. GWAS have the potential not only to facilitate risk prediction but also to provide novel insights into disease mechanisms [99]. However, the HapMap focuses on nuclear DNA, and, to date, there have been no genome-wide association studies of mtDNA and breast cancer risk. However, such studies are warranted in light of the fact that small-scale studies that have tested a limited number of mtDNA polymorphisms have provided some support for a role for mtDNA variants in influencing breast cancer risk. Given that most associations identified through genome-wide studies do not involve previous candidate genes, the results of genome-wide studies may immediately suggest new biological hypotheses [99] and provide a basis for functional studies. In view of the putative role of oxidative stress in carcinogenesis, and of the mitochondria as a major source of ROS production, such studies have the potential to provide valuable insights into the role of the mitochondria in the etiology of breast cancer. 

In order to investigate the pathogenic significance of germline variants and somatic mtDNA alterations, additional functional studies of the effects of alterations in the mitochondrial genome of cancer cells are required.

## 12. Interplay between Mitochondrial and Nuclear DNA Variants

 In addition to focusing on the association of polymorphisms in mitochondrial DNA with breast cancer risk, the interplay between mitochondrial DNA variants and nuclear DNA variants also merits examination. For example, the subunits of complex II of the respiratory chain complex are encoded entirely by nuclear genes, and three of these genes have been shown to be tumor suppressors [118]. Furthermore, mitochondrial DNA synthesis, replication, transcription, and translation are under nuclear control [119], and nuclear-mitochondrial communication disorders have been described, which result in a loss of integrity of the mitochondrial genome [118]. Therefore, if genome-wide association studies uncover genetic variants associated with breast cancer risk, a next step would be to conduct studies focusing on nuclear DNA (nDNA) variants that encode for mitochondrial proteins, to examine both the association between these variants and breast cancer risk and the interaction between nDNA and mtDNA variants in relation to risk.

## 13. Conclusion

To date, most studies examining the role of mitochondrial damage in carcinogenesis have focused on mtDNA somatic mutations. In view of the putative role of oxidative stress in carcinogenesis, and of the mitochondria as a major source of ROS production, the role of mitochondrial DNA variants in the etiology of breast cancer represents a potentially promising area of study. Genome-wide association studies are likely to identify new mtDNA variants which modify breast cancer risk. In addition to assessing the main effects of specific variants, gene-gene and gene-environment interactions are likely to explain a greater proportion of the variability in breast cancer risk. The results of such studies might have translational potential, given that they may provide insight into the biological processes underlying breast cancer development, and, hence, suggest strategies for prevention and therapy [99].

## Figures and Tables

**Table 1:** Association studies of mtDNA variants and breast cancer risk.

Reference	Study subjects*	Source of study subjects	MtDNA variant (s)	OR (95% CI)**
Canter et al. [24]	48 AA cases, 54 AA controls (USA)	Hospital-based	G10398A	2.90 (0.61–18.3)
	654 AA cases, 605 AA controls (USA)	Population-based	G10398A	1.60 (1.10–2.31)
	879 White cases, 760 White controls (USA)			1.03 (0.81–1.31)

Canter et al. [92]	AA subjects as in Canter et al. (2005)		T4216C*G10398A	3.31(1.07–10.25)

Darvishi et al. [94]	124 cases, 273 ethnically matched controls (India)	Details not provided	G10398A	1.73 (1.13–2.66)

Bai et al. [91]	156 non-Jewish European-American cases, 260 non-Jewish European-American controls (USA)	Cases referred to Molecular Genetics Laboratory for BRCA1/2 testing; controls were individuals referred for genetic testing	69 variants tested.	
			Significant results:	
			G9055A	3.03 (1.63–5.63)
			A10398G	1.79 (1.14–2.81)
			T16519C	1.98 (1.25–3.12)
			T3197C	0.31 (0.13–0.75)
			G13708A	0.47 (0.24–0.92)
			Haplotype K	3.30 (1.63–5.63)
			Haplotype U	0.37 (0.19–0.73)

Mosquera-Miguel et al. [95]	464 cases, 453 ethnicity-matched controls (continental Spain), 302 cases, 295 ethnicity-matched controls (Canary Islands)	Details not provided	25 variants tested	None of the variants was associated with altered risk in either study after adjustment for multiple testing

Covarrubias et al. [36]	Same subjects as in Bai et al. [91]		17 variants tested for all possible 2-way interactions	A10398G*A12308G (*P* = .004) All other interactions NS after control for FWER

Setiawan et al. [97]	542 AA cases, 282 AA controls (USA)	Population-based	G10398A	1.73 (0.87–3.47)
	391 AA cases, 460 AA controls (USA)	Multiethnic cohort	G10398A	1.08 (0.62–1.86)
	524 AA cases, 236 AA controls (USA)	Population-based	G10398A	0.81 (0.43–1.51)

Ye et al. [98]	1058 Chinese cases, 1129 Chinese controls (China)	Population-based	D-loop (CA)_n_ repeat polymorphism:	
			(CA)_5_	1.00 (reference)
			(CA)_4_	1.02 (0.85–1.21)
			(CA)_6_	0.84 (0.50–1.41)
			(CA)_7_	0.50 (0.27–0.93)
			(CA)_8–11_	1.59 (0.64–3.91)

Czarnecka et al. [93]	44 Polish cases, 100 Polish controls (Poland)	Clinic-based cases, population-based controls	A10398G	9.51(2.64–33.88)

Pezzotti et al. [96]	1561 cases, 2209 controls in Nurses' Health Study; 678 cases, 669 controls in Women's Health Study	Population-based	A10398G	
			Nurses' Health Study	1.01 (0.85–1.19)
			Women's Health Study	0.94 (0.72–1.22)

*AA = African-American; NS = not significant; FWER = familywise error rate

**Canter et al. [24] estimates are crude estimates—adjustment for other factors in population-based component did not change them; Darvishi et al. [94] estimates are crude; Bai et al. [91] *P*-values adjusted for familywise error rate.
